# Intérêt de l’imagerie dans les tumeurs osseuses bénignes chez l’enfant

**DOI:** 10.11604/pamj.2016.24.179.9920

**Published:** 2016-06-30

**Authors:** Ousmane Traoré, Kamilia Chban, Alzavine Fleur Hode, Yaya Diarra, Siham Salam, Lachen Ouzidane

**Affiliations:** 1 Service de Radiologie Pédiatrique, Hôpital d’enfant CHU Ibn Rochd, Casablanca, Maroc

**Keywords:** Tumeur, os, bénigne, imagerie, enfant, Tumor, bone, benign, imagery, children

## Abstract

Les tumeurs osseuses bénignes sont beaucoup plus fréquentes que les tumeurs malignes en milieu pédiatrique. L’exostose (ostéchondrome) en est la plus fréquente. Les différentes techniques d'imagerie occupent une place déterminante dans l'étude de ses tumeurs notamment la radiographie standard. Le but de ce travail est de souligner l’intérêt de l’imagerie dans la prise en charge diagnostique des tumeurs bénignes osseuses chez l’enfant à travers une étude rétrospective portant sur 169 patients. Tous ces patients ont été explorés par la radiographie standard, un complément scanner avec reconstruction multiplanaires avant et après injection de PDC et/ouune IRM 1.5 Tesla a été réalisé en fonction de l’indication. L’âge moyen est de 6 ans avec une légère prédominance masculine. Cliniquement, la tuméfaction est présente dans 35% des cas. La douleur dans 29 %des cas. La localisation la plus fréquente est la métaphyse sur les os long: fémur: 25% des cas, humérus: 17 % des cas, tibia: 21% des cas. Les principales tumeurs bénignes retrouvées sont l’exostose (20,12 %), le kyste osseux (31,95%) et l’ostéoblastome (16, 57%). L’imagerie permet de préciser la topographie et l’extension de la lésion dans l’os, apporter des arguments en faveur de la bénignité et parfois, en faveur de l’origine de la lésion. La radiographie standard seule permet souvent de poser un diagnostic de certitude dans certains cas.

## Abstract (en)

Benign bone tumors are more common than malignant tumors in pediatrics. The exostosis (ostéchondrome) is the most common. The different imaging techniques are pivotal in the study of tumors including its standard radiography. The aim of this work is to highlight the interest in imaging the diagnostic management of bone benign tumors in children through a retrospective study of 169 patients. All patients were investigated by plain radiography, scanner supplement with multiplanar reconstruction before and after injection of PDC and / or MRI 1. 5 Tesla was performed according to the indication. The average age is 6 years with a slight male predominance. Clinically, the swelling is present in 35% of cases. The pain in 29% of cases. The most common location is the metaphyseal long bone on: Femur: 25% of cases, humerus: 17% of cases, Tibia: 21% of cases. Main Benign tumors are found exostosis (20. 12%), bone cyst (31. 95%) and osteoblastoma (16, 57%). The imagery is used to specify the topography and extension of the lesion in the bone provide arguments in favor of benign and sometimes in favor of the cause of the injury. The only standard radiograph often provides a diagnosis of certainty in some cases.

## Introduction

Les tumeurs osseuses bénignes sont beaucoup plus fréquentes que les tumeurs malignes chez l’enfant. Les différentes techniques d´imagerie occupent une place déterminante dans l´étude de ces tumeurs car les types histologiques sont très variés [1–3]. L’analyse sémiologique rigoureuse de la radiographie standard associée à la synthèse clinique permet parfois de poser le diagnostic avec certitude [4]. Les examens comme le scanner (TDM), l’imagerie par raisonace magnétique (IRM) et l’échographie sont réalisés en seconde intention suite à la radiographie standard [5]. L’aspect radiographique typique de certaines lésions permet d’éviter la biopsie. Le but de ce travail est d’apporter l’intérêt de l’imagerie dans la prise en charge des tumeurs bénignes osseuses chez l’enfant.

## Méthodes

Il s’agit d’une étude rétrospective portant sur 169 patients reçus au service de Radiologie Pédiatrique de l’Hôpital d’enfant du CHU Ibn Rochd de Casablanca pendant une période de 4 ans. Les examens réalisés sont: la radiographie standard chez 148 enfants; le scanner 16 barrettes avec reconstruction multiplanaires avant et après injection de PDC chez 21 patients et l’IRM 1.5 Tesla chez 1 patient.

**Critères d’inclusion:** les patients ayant réalisé dans le cadre de leur bilan au moins un des trois examens d’imagerie (radiographie standard, scanner ou IRM).

## Résultats

**Figure 1:**
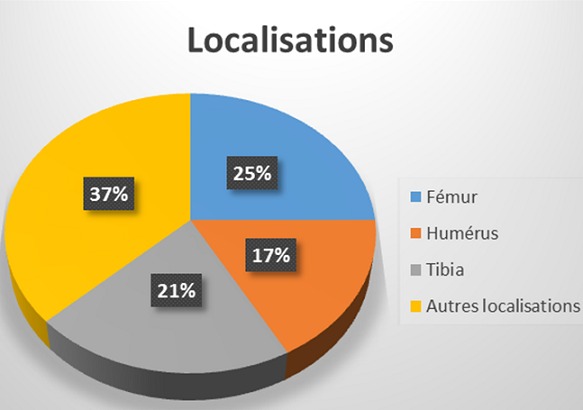
Siège des lésions

**Figure 2:**
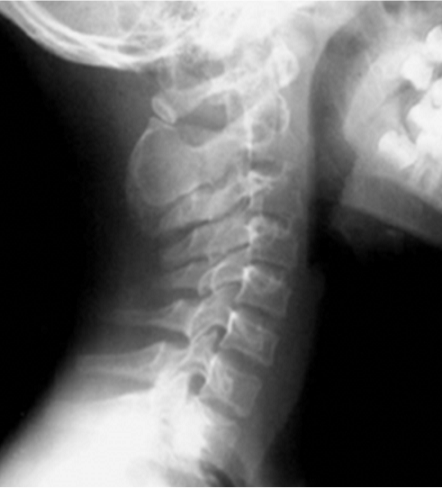
Radiographie du rachis cervical profil, formation ostécondensante au niveau du corps vertébral de C2 évoquant un ostéoblastome

**Figure 3:**
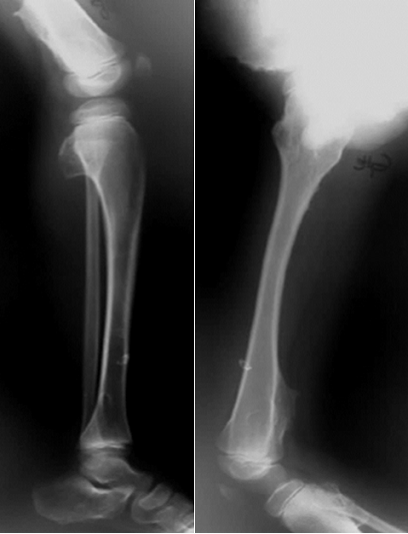
Radiographie des MI, formations osseuses xérophytiques en continuité avec le cortex de la partie proximal du tibia à gauche et de la région métaphysaire inférieure du fémur: exostose

**Figure 4:**
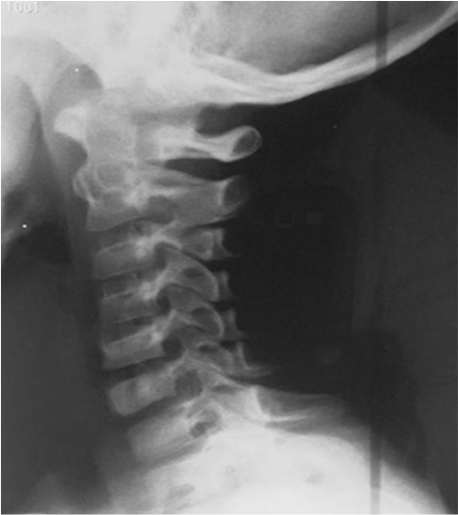
Radiographie du rachis cervical profil d’une patiente de 7 ans: interprétée normale

**Figure 5:**
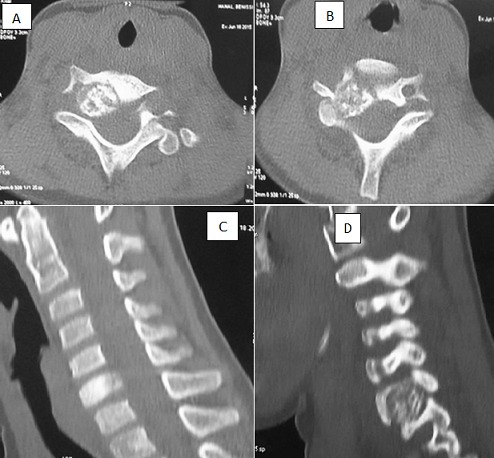
TDM en reconstruction axiale (A-B) et sagittale (C-D) en fenêtre osseuse de la même patiente de 7 ans objective une formation arrondie, bien limité siège d’ostéolyse et d’ostéocondensation du corps de C7 latéralisé à droite rétrécissant le trou de conjugaison de C7-D1 et le canal médullaire en regard arrive au contact du pédicule droit non vue à la radiographie: ostéoblastome

**Figure 6:**
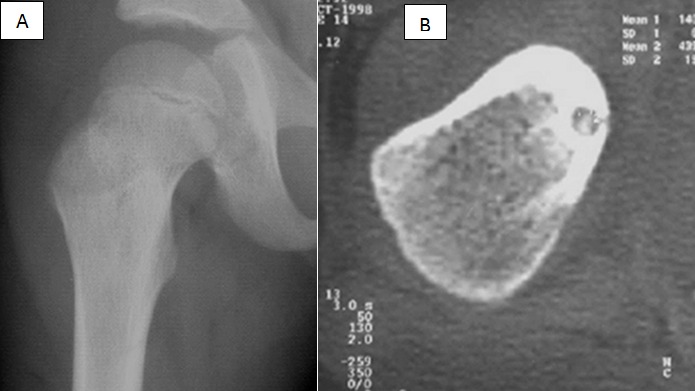
Ostéome ostéoïde, radiographie (A), ostéocondensation du col fémoral TDM (B): image typique du nidus

**Figure 7:**
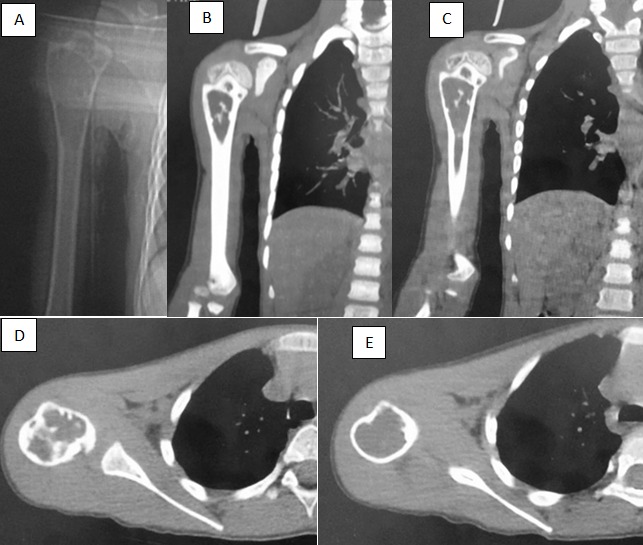
Radiographies (A) et scanner (B,C,D,E) montrant une lacune ovoïde centrée de contours nets avec pseudo cloisons par crêtes pariétales de densité liquidienne au niveau métaphysaire étendu sur la diaphyse humérale droite: kyste osseux essentiel Confirmation histologique: kyste osseux essentiel atypique

L’âge moyen est de 6 ans avec une légère prédominance masculine(89sexe masculin/80sexe féminin). Les signes cliniques étaient dominés par la tuméfaction des parties molles dans 35% des cas, la douleur dans 29 %des cas, la fracture pathologique (19 % des cas) et 17 % des tumeurs sont de découverte fortuite. Le siège le plus fréquent des lésions est métaphysaire sur les os long: Fémur (siège majoritaire à 25% des cas); Humérus (17 % des cas); Tibia (21% des cas) (Figure 1). La radiographie standard a retrouvé les aspects suivants: lésions ostéolytiques dans 150 cas; les lésions ostécondensantes dans 30 cas et de réaction périostée uni lamellaire dans 5 cas (Figure 2, Figure 3). Cette technique radiographique a été suffisante pour évoquer le diagnostic sans faire recours aux autres moyens d’imagerie dans 80 cas soit 54% des cas. 15% d’exostose; 12% d’ostéoblastome; 12% de kyste osseux essentiel; 11% de kyste osseux anévrismal et 4% de chondrome myxoïde Le scanner objective des lésions multiples ostéolytiques géographiques, lacunaires siège de cloisons, de trabéculations ainsi que des zones liquidiennes dans certains cas avec des ostéocondensations. L’envahissement des parties molles a été retrouvé dans 1 cas, et la rupture corticale dans 5 cas, tous représentés dans le Tableau 1, et les Figure 4, Figure 5, Figure 6, Figure 7). Les principales tumeurs bénignes retrouvées sont l’exostose d’origine ostéogénique représentant (20,12 %) etl’ostéoblastome (16, 57%); ainsi que les pseudotumeurs représentées ici par le kyste osseux essentiel (17, 75 %) et le kyste osseux anévrysmal (14,20%). Il y avait également des lésions d’origine cartilagineuses; d’origine fibroblastiques et des tumeurs histiocytaires (Tableau 2).

**Tableau 1:** Anomalies scanographiques

Sémiologie TDM	Effectifs	Pourcentage
**Ostéolyse**	**15**	**71,43**
Ostéocondensation	8	38,09
Réactions périostées	2	9,52
**Rupture corticale**	**5**	**23,81**
Envahissement des parties molles	1	4,76

**Tableau 2:** Répartition des différents types histologiques

Types histologiques	Effectifs	Pourcentage
**Exostose**	**34 cas**	**20,12**
**Kyste osseux essentiel (KOE)**	**30 cas**	**17,75**
**Kyste osseux anévrysmal (KOA)**	**24 cas**	**14,20**
Ostéome ostéoïde (OO)	15 cas	8,88
Maladie exostosante (ME)	13 cas	7,69
**Ostéoblastome**	**28 cas**	**16,57**
Chondrome	8 cas	4,73
Granulome éosinophile (GE)	6 cas	3,55
Fibrome non ossifiant (FNO)	4 cas	2,37
Chondromatose	3 cas	1,78
Maladie d’Ollier	1 cas	0,59
Fibrome chondromyxoïde (FCM)	1 cas	0,59
Chondroblastome	1 cas	0,59
Tumeur à cellules géantes (TCG)	1 cas	0,59
**Total**	**169 cas**	**100**

## Discussion

Les tumeurs osseuses bénignes de l´enfant sont variées; avec différents types histologiques. Elles représentent 6 à 10% des tumeurs de l’enfant [1–6]. Les tumeurs osseuses bénignes sont beaucoup plus fréquentes que les tumeurs malignes. L’exostose (ostéchondrome) est la plus fréquente des tumeurs bénignes [7, 8] comme fut dans notre serie. Elle atteint les adolescents au tour de l’âge de 15 ans [9]. Les différentes techniques d´imagerie occupent une place déterminante dans l´étude de ces tumeurs.

**Les différents types de tumeurs osseuses benignes et leurs origines** [10–13]: selon leur origine on distingue: les tumeurs cartilagineuses (Ostéochondrome, chondrome, chondroblastome bénin) 12 cas dans notre serie; Les tumeurs ostéogéniques (Ostéome osteoide, Ostéoblastome, Ostéome) 90 cas retrouvés dans notre étude; Les tumeurs fibroblastiques( FNO, Dysplasie fibreuse) 4 cas dans notre serie; Les tumeurs histiocytaires( Histiocytose Langerhansienne / granulome éosinophile, Tumeur à cellules géantes) 7 cas dans notre serie dont 6 graniulome éosinophile et 1 cas de tumeur a cellule geante; Les tumeurs vasculaires: angiome ( adulte de 40-50) aucun cas retrouvés dans notre serie; Les Pseudo tumeurs(Kyste essentiel, kyste anévrisme) 31,95 cas dans notre étude et divers regroupant l’ énostose (îlot condensant bénin), et desmoid périosté dont aucun cas n’a été retrouvé dans notre etude. Les aspects cliniques: Les signes cliniques sont non spécifiques. Il peut s’agir d’une tuméfaction des parties molles, 35% des cas retrouvés dans notre serie, d’une douleur, et/ou d’une fracture pathologique. Dans certains cas, le diagnostic est découvert de façon fortuite [14].

**Aspect radiologique:** la localisation fréquente de tumeurs osseuses bénignes est métaphysaire sur les os long [15, 16] qui concorde avec notre étude ( 25% des cas sur le fémur, 17% sur le l’humérus et 21 sur le tibia).

**La sémiologie radiologique des tumeurs osseuses bénignes [16–20]: les** signes radiologiques orientant vers le diagnostic d’une tumeur bénigne sont: L’Ostéolyse; La petite taille de la lésion (<6 cm); Les limites nettes; Le liseré de condensation périphérique; L’intégrité des corticales ( soufflée+) sauf fracture; L’absence d’envahissement des parties molles; La réaction périostée absente ou compacte; La stabilité par rapport aux clichés antérieurs; La matrice tumorale: permet d’approcher la nature osseuseou cartilagineuse de la lésion.

**La radiographie standard:** elle est réalisée en première intention dans l’exploration des lésions osseuses. On réalise deux incidences orthogonales: face et profil. Cette radiographie permet de poser le diagnostic dans la plupart des cas. Elle constitue une base à l’orientation étiologique. Elle peut être suffisante pour le diagnostic de certaines tumeurs: kyste osseux essentiel; exostose; chondrome multiples et dysplasie fibreuse. Parfois, elle oriente d’autres explorations.

**L’échographie:** elle est indiquée par la radiographie standard. Elle est réalisée à l’aide d’une sonde superficielle 7 à 12 Mhz et permet l’exploration des tissus mous autour de la lésion osseuse.

**Le scanner :** la TDM permet une meilleure analyse du contenu tumoral et des os difficiles à évaluer sur le cliché standard en particulier les os courts et les os plats. Si les éléments radiographiques sont suffisants pour affirmer le diagnostic, la réalisation du scanner n’est pas obligatoire.

**L’Imagerie par raisonnance magnétique:** l’IRM n’est pas nécessaire dans l’exploration des tumeurs bénignes osseuses. Lorsqu’elle est faite, elle permet de déterminer avec précision les limites de la tumeur et ses rapports avec les structures adjacentes.

**La scintigraphie:** elle est utile pour apprécier la vascularisation tumorale et la réaction métabolique osseuse. Sa spécificité est faible sauf dans l’ostéome ostéoïde [17, 19, 20].

**Anatomopathologie:** certaines tumeurs bénignes ou dystrophie pseudo-tumorales ne justifient pas d’une biopsie. C’est le cas d’une exostose à fortiori si elle rentre dans le cadre d’une maladie exostosante, de la maladie des chondromes multiples, de la forme polyostotique de dysplasie fibreuse ou du kyste essentiel d’aspect radiologique typique. Une biopsie osseuse avec examen anatomopathologique peut s’avérer nécessaire pour établir le diagnostic d´une tumeur osseuse lorsque les critères radiologiques ne sont pas spécifiques [20].

## Conclusion

L’imagerie permet de préciser la topographie et l’extension de la lésion dans l’os, d’apporter des arguments en faveur de la bénignité et parfois, en faveur de l’origine de la lésion. La radiographie standard seule permet souvent de poser un diagnostic de certitude. Elle permet la mise en évidence de la lésion, sert de base à l'orientation étiologique, et à l’indication des autres explorations. Le scanner permet l’étude de l’os cortical et/ou trabéculaire. L’imagerie par raisonace magnétique permet de faire l’exploration des tissus mous adjacents et du canal rachidien. L’échographie permet l’exploration des tissus mous autour de la lésion.

### Etat des connaissances actuelles sur le sujet

- C’est une tumeur plus fréquente en milieu pediatrique que chez l’adulte et aussi plus fréquente que les tumeurs malignes osseuses.

### Contribution de notre étude à la connaissance

- La particularité de la prise en charge diagnostique fiable en imagerie se limité a des moyens d’imagerie moins irradiant et moins couteux dans certains cas.

## References

[1] Ambrosi R, Barbato A, Caldarini C, Biancardi E, Facchini RM (2016). Gradual Ulnar Lengthening In Children With Multiple Exostoses And Radial Head Dislocation: Results At Skeletal Maturity. J Child Orthop.

[2] Cho YJ, Jung Yonsei ST (2014). Gradual Lengthening Of The Ulna In Patients With Multiple Hereditary Exostoses With A Dislocated Radial. Head Med J.

[3] Bair HJ, Schmitt R, Moos P, Fellner F, Dvorak O, Rupprecht H, Lenz M (1997). Malignant Transformation In Multiple Cartilaginous Exostoses Diagnostic Value Of Magnetic Resonance Tomography. J Child Orthop.

[4] Gennari JM, Themar-Noel C, Panuel M, Bensamoun B, Deslandre C, Linglart A, Sokolowski M, Ferrari A (2015). Adolescent Spinal PaIn: The Pediatric Orthopedist's Point Of View, French Society of Spine Surgery (SFCR). Orthop Traumatol Surg Res.

[5] Traore SY, Dumitriu DI, Docquier PL (2014). Intra-Articular Osteoid Osteoma Mimicking Juvenile Arthritis. Case Rep Orthop.

[6] Cerase A, Priolo F (1998). Skeletal Benign Bone-Forming Lesions. Eur J Radiol.

[7] Gokce E, Ayan E, Celikyay F, Acu B (2013). Radiological Imaging Findings Of A Case With Vertebral Osteoid Osteoma Leading To Brachial Neuralgia. J Clin Imaging Sci.

[8] Assoun J, De Haldat F, Richardi G, Billey T, Dromer C, Fournié B, Bonnevialle P, Railhac JJ (1993). Magnetic Resonance Imaging In Osteoid Osteoma. Rev Rhum Ed Fr.

[9] Spouge AR, Thain LM (2000). Osteoid Osteoma: Mr Imaging Revisited. Clin Imaging.

[10] Atesok KI, Alman BA, Schemitsch EH, Peyser A, Mankin H (2011). Osteoid Osteoma And Osteoblastoma. J Am Acad Orthop Surg.

[11] Youssef BA, Haddad MC, Zahrani A, Sharif HS, Morgan JL, al-Shahed M, al-Sabty A, Choudary R (1996). Osteoid Osteoma And Osteoblastoma: Mri Appearances And The Significance Of Ring Enhancement. Eur Radiol.

[12] Woertler K (2003). Benign Bone Tumors And Tumor-Like Lesions: Value Of Cross-Sectional Imaging. Eur Radiol.

[13] Smadhi H, Ben Hamad W, Neffati O, Kammoun H, Braham E, Fekih L, Megdiche ML (2015). A Rare Cause Of A Posterior Mediastinal Mass. Rev Pneumol Clin.

[14] Bashaireh KM (2016). Patellar Subluxation With Early-Phase Synovial Chondromatosis Of The Knee. Orthopedics.

[15] El Rassi G, Matta J, Hijjawi A, Khair OA, Fahs S (2015). Extra-Articular Synovial Chondromatosis Eroding And Penetrating The Acromion. Arthrosc Tech.

[16] Ropars M, Hervé A, Stock N, Guillin R, Guggenbuhl P (2015). Giant Synovial Chondromatosis Of The Metacarpophalangeal Joint. Joint Bone Spine.

[17] Saibaba B, Sudesh P, Govindan G, Prakash M (2015). Pediatric Subtalar Joint Synovial Chondromatosis Report Of A Case And An Up-To-Date Review. J Am Podiatr Med Assoc.

[18] Sedeek SM, Choudry Q, Garg S (2015). Synovial Chondromatosis Of The Ankle Joint: Clinical, Radiological, And Intraoperative Findings. Case Rep Orthop.

[19] Shaw A, Zibly Z, Prasad V, Ikeda D, Boue D, Governale LS (2014). Synovial Chondromatosis Of The Cervical Spine: A Case Report And Review Of The Literature. Pediatr Dev Pathol.

[20] Balasundaram A, Geist JR, Gordon SC (2009). Klasser GD:Radiographic Diagnosis Of Synovial Chondromatosis Of The Temporomandibular Joint: a case report. J Can Dent Assoc.

